# Metagenomic mining reveals extensive novelty, enhanced biodegradation potential, and untapped biosynthetic capacity in Chinese oilfield microbiomes

**DOI:** 10.1128/aem.00392-26

**Published:** 2026-04-01

**Authors:** Changhao Zhou, Shuoliang Wang, Hui Zhao, Shiqi Wang, Liangliang Jiang, Chunlei Yu

**Affiliations:** 1 School of Energy Resources, China University of Geosciences (Beijing) 113084 , Beijing, China; 2 Shandong Zhongdi Yicai Petroleum Technology Co., Ltd., Dongying, Shandong, China; 3 School of Petroleum Engineering, Yangtze University, Wuhan, Hubei, China; 4 Department of Chemical and Petroleum Engineering, University of Calgary 2129, Calgary, Alberta, Canada; Colorado School of Mines, Golden, Colorado, USA

**Keywords:** oil reservoirs, metagenome, AMP, biodegradation

## Abstract

Oil reservoir microorganisms represent a vast and largely unexplored reservoir of biological diversity and functional potential, yet comprehensive studies on their genomic and metabolic characteristics remain limited. To address this gap, we collected 101 metagenomic sequencing samples from 13 distinct oilfields across China. Through extensive *de novo* assembly and binning processes, we successfully reconstructed 3,057 medium and high-quality metagenome-assembled genomes (MAGs), providing an unprecedented genomic resource for reservoir microbiome research. Strikingly, 73.77% of these MAGs correspond to novel taxa at the species level, highlighting the significant unexplored microbial diversity in these environments. Detailed genomic analysis revealed that MAGs classified under the class Planctomycetia exhibited notably larger genome sizes, primarily driven by the expansion of specific gene families, suggesting adaptive evolutionary strategies in hydrocarbon-rich environments. Furthermore, we identified 68 genes implicated in anaerobic alkane biodegradation pathways, with samples from the Shengli oilfield demonstrating particularly enhanced biodegradation potential, indicating site-specific functional adaptations. Beyond biodegradation, our study uncovered three MAGs assigned to the genus *Tistrella*, which harbored a remarkable abundance of biosynthetic gene clusters (BGCs) for secondary metabolites. Additionally, 14 candidate antimicrobial peptides (cAMPs) were detected, signifying the potential for novel bioactive compound discovery. Critically, both the *Tistrella* MAGs and cAMPs were identified for the first time within petroleum reservoir ecosystems, underscoring the unique biotechnological value of these environments. This research not only expands our understanding of oil reservoir microbial communities but also emphasizes their substantial implications for industrial applications, including bioremediation, antimicrobial development, and sustainable resource management.

**IMPORTANCE:**

This study provides a groundbreaking genomic exploration of oil reservoir microbiomes across 13 Chinese oilfields, reconstructing 3,057 medium and high-quality metagenome-assembled genomes (MAGs). Remarkably, 73.77% of these MAGs represent novel species, revealing vast unexplored microbial diversity. We observed genome expansion in Planctomycetia lineages and identified 68 genes involved in anaerobic alkane degradation, with heightened biodegradation potential in Shengli oilfield samples. Crucially, we discovered three *Tistrella* MAGs rich in biosynthetic gene clusters (BGCs) for secondary metabolites and 14 candidate antimicrobial peptides (cAMPs), both reported for the first time in petroleum reservoirs. These findings highlight the immense biotechnological potential of reservoir microbiomes, offering new pathways for bioremediation strategies in oil-contaminated environments and novel sources for antimicrobial discovery. This work underscores the critical need for continued investigation into these unique ecosystems to harness their functional capabilities for energy sustainability and pharmaceutical innovation.

## INTRODUCTION

Petroleum is a complex mixture consisting of alkanes, aromatic hydrocarbons, and compounds containing carbon, hydrogen, oxygen, sulfur, and nitrogen. Nevertheless, a large number of microorganisms can survive and exert various functions in reservoir environments. Microbial-enhanced oil recovery (MEOR) is a promising tertiary oil recovery technology that has attracted extensive attention from the global petroleum industry in recent years due to its advantages of environmental friendliness, simple operation, and high cost-effectiveness (1). In the MEOR process, cultured microorganisms or nutrient solutions are injected into underground oil reservoirs, where the microorganisms grow, reproduce, and release specific metabolites to improve oil recovery efficiency (2, 3). Based on different operation modes and injected microbial strains, MEOR technology is mainly classified into five types: microbial huff and puff recovery (MHPR), microbial flooding recovery (MFR), microbial selective plugging recovery (MSPR), biopolymer flooding recovery (BFR), and microbial wax removal and control (MWRC) (4). MHPR and MFR mainly utilize microbial metabolites to improve fluid properties (e.g., reducing crude oil viscosity and oil-water interfacial tension) and thus promote oil migration (5, 6). MWRC enhances oil production efficiency by degrading crystalline wax deposited in oil well bores and gathering pipelines (7).

In the three aforementioned MEOR technologies, microorganisms primarily utilize long-chain alkanes as carbon sources for metabolism; thus, the biodegradation of long-chain n-alkanes is a common phenomenon in petroleum reservoirs (8). Decades of research have confirmed that hydrocarbon-degrading bacteria employ alkylsuccinate synthase (AssA) or benzylalkylsuccinate synthases (BssA) to catalyze the fumarate addition reaction of alkanes, generating alkylsuccinates under anaerobic conditions, with nitrate, iron, or sulfate serving as electron acceptors (9–12). In the absence of exogenous electron acceptors, syntrophic interactions between bacteria and archaea drive the biodegradation process. Bacteria first cleave long-chain alkanes into simple compounds (e.g., acetate and formate), benzoyl-CoA reductase subunits B (BcrB) and C (BcrC) were used in this system, and methanogenic archaea subsequently convert these compounds into methane via methyl-coenzyme M reductase (Mcr) (13, 14). Recent studies have revealed that archaeal alkyl-coenzyme M reductase (Acr), a variant of Mcr, can directly activate long-chain alkanes (15). Laso-Pérez (16) identified both Acr and Mcr genes in the metagenome-assembled genomes (MAGs) of Methanoliparia. Subsequent research demonstrated that *Methanoliparum* archaea can independently activate long-chain alkanes (C^13+^) and alkyl-substituted hydrocarbons by utilizing Acr variants (17).

Beyond MEOR, researchers have also shown great interest in other functions of reservoir microorganisms, such as anaerobic ammonium oxidation (anammox) (18) and post-contamination remediation (19). These studies are mostly focused on solving practical problems in oil reservoir production, while the high biological value of the microorganisms themselves remains to be explored in depth. For instance, with the rapid development of deep learning, an increasing number of bioactive peptides have been identified from microorganisms in various environments. Antimicrobial peptides (AMPs) are a class of short peptides with broad-spectrum biological activity, generally consisting of fewer than 50 amino acids. In microorganisms, they are mainly produced via two secondary metabolic pathways, ribosomally synthesized and post-translationally modified peptides (RiPPs) and nonribosomal peptide synthetases (NRPSs), and exhibit antibacterial, antifungal, antiviral, and even anticancer activities (20). Ma (21) identified 2,349 candidate AMPs (cAMPs) from the human gut microbiome using Attention, LSTM, and BERT models, among which 181 exhibited antibacterial activity. Chen (22) discovered 121 marine-derived cAMPs, with 10 showing potent biological activity. Santos-Júnior (23) constructed a global database containing more than 860,000 peptides from 150,000 microbial genomes and verified the antimicrobial efficacy of 79 out of 100 tested cAMPs. Notably, AMPs derived from petroleum microbiomes remain largely unexplored.

In this study, we collected 101 publicly available metagenomic samples from major Chinese oilfields, conducted genome assembly and binning, investigated the core microbial taxa and interfield differences in microbial community structure, characterized the alkane biodegradation potential of microbial communities, and simultaneously mined potential AMPs from these samples. This work lays a foundation for further exploitation and utilization of petroleum microbial resources.

## MATERIALS AND METHODS

### Data collection

Using the keywords “Metagenome AND China oil reservoirs,” we searched PubMed for articles published between 2018 and 2025. A total of 101 metagenomic samples from 13 Chinese oilfields were collected (Table S1), and raw sequencing data were downloaded via links provided in the publications (24–29).

### Metagenomic assembly, binning, and quality control

Raw sequencing data in SRA format were converted to FASTQ using SRA Toolkit (v3.1.1). Adapters and low-quality sequences were removed with SOAPnuke (v1.6.5) (30). Clean reads were assembled using MEGAHIT (v1.1.3) (31) with the following parameters: --min-count 2 --k-min 33 --k-max 83 --k-step 20 --no-mercy. A total of 101 assemblies were binned via MetaWRAP (v1.3.2) (32) using MetaBAT2, MaxBin2, and CONCOCT. Bins from all three tools were integrated with bin_refinement, retaining those with ≥50% completeness and ≤10% contamination, yielding 5,007 medium- and high-quality bins (33). These bins were clustered at 95% average nucleotide identity (ANI) using dRep (v3.2.2) (34) with the following parameters: -comp 50 -con 10 -pa 0.9 --S_ani 0.95 --cov_thresh 0.3 --strain_heterogeneity_weight 0, resulting in 3,057 representative MAGs for downstream analysis.

### Taxonomic annotation and relative abundance calculation

The 3,057 MAGs were taxonomically annotated using GTDB-Tk (v2.4.1) (35) with default parameters (data set: r226). Bacterial and archaeal phylogenetic trees were constructed from aligned protein sequences (generated by GTDB-Tk) using IQ-TREE (v2.1.4) (36) and visualized with iTOL (v6.0) (37).

For relative abundance calculation, MAGs were merged into a reference genome database. Clean reads from all 101 samples were aligned to this database using Bowtie2 (v2.2.5) (38) with the following parameters: -D 10 -R 2 -N 1 -L 22 -i S,0,2.50. SAM files were converted to sorted BAM files using samtools (v1.9) (39), followed by filtering of paired-end reads with >97% similarity using BBMap (v39.33) (40) (reformat.sh minidfilter = 0.97 primaryonly = t pairedonly = t). Relative abundance was computed with CoverM (v0.7.0) (41), retaining reads covering ≥60% of the genome (coverm genome --proper-pairs-only --min-read-percent-identity-pair 0.97 --min-covered-fraction 0.6). Alpha diversity analysis and visualization were performed using OmicStudio (42).

### Coding gene annotation and orthogroup identification

Open reading frames (ORFs) from the 3,057 MAGs were predicted with Prokka (v1.14.6) (43), identifying 8,514,112 coding genes. Functional annotation was performed using eggNOG-mapper (v2.1.12) (44) against eggNOG DB (v5.0.2). Orthogroups (OGs) for 103 *Planctomycetia* and *Phycisphaerae* MAGs were identified with OrthoFinder (v2.5.5) (45) using the following parameters: -a 2 -M msa -S diamond -A muscle -T fasttree -og.

### Anaerobic hydrocarbon biodegradation gene mining

Hidden Markov models (HMMs) for AcrA genes were downloaded from PFAM (PF02249 for MCR_alpha; PF02745 for MCR_alpha_N). AssA reference sequences were obtained from the AnHyDeg database (https://github.com/AnaerobesRock/AnHyDeg), and HMM profiles were built using hmmbuild (HMMER v3.1b2) (46). BcrB and BcrC references were identified via Annotree (47) (TIGR02260 and TIGR02263) and converted to HMMs. Candidate genes were screened by aligning coding proteins to HMMs using hmmsearch (e-value<1e−5), followed by deduplication at 99% sequence similarity with CD-HIT (v4.8.1) (48).

AcrA reference sequences were downloaded from KEGG (K00399). Reference sequences were merged with deduplicated candidate gene sequences, aligned with MUSCLE (v3.8.31) (49), and filtered via maximum-likelihood phylogeny using IQ-TREE (-m WAG -bb 1000).

### cAMP identification

Biosynthetic gene clusters (BGCs) were predicted using antiSMASH (v7.1.0) (50) with the following parameters: -c 8 --taxon bacteria --genefinding-tool prodigal --cb-general --cb-subclusters --cb-knownclusters --asf --pfam2go. Core peptides from RiPP-class BGCs were screened following Chen (22) and scored using Attention, LSTM, and BERT models. Peptides with scores > 0.5 across all models were classified as cAMPs (21, 22). Briefly, the scripts provided by Chen (22) were first used to extract core peptides from RiPP-class BGCs. Subsequently, these core peptides were converted from FASTA format into a format compatible with the Attention and LSTM models, whereas the BERT model continued to use the FASTA format. Next, the pretrained Attention, LSTM, and BERT models were downloaded, and each of these three models was used to score the core peptides individually. Finally, the results from the three models were integrated, and only those core peptides with a score > 0.5 in all models were defined as cAMPs. All the aforementioned scripts and pretrained models are available in the study by Chen (22).

### Statistics

All statistical significance tests used in this study were *t*-tests by R package “ggpubr,” and all correlation coefficients employed were Pearson correlation coefficients.

## RESULTS

### Overview of genome catalogs in China oil reservoirs

**Fig 1:**
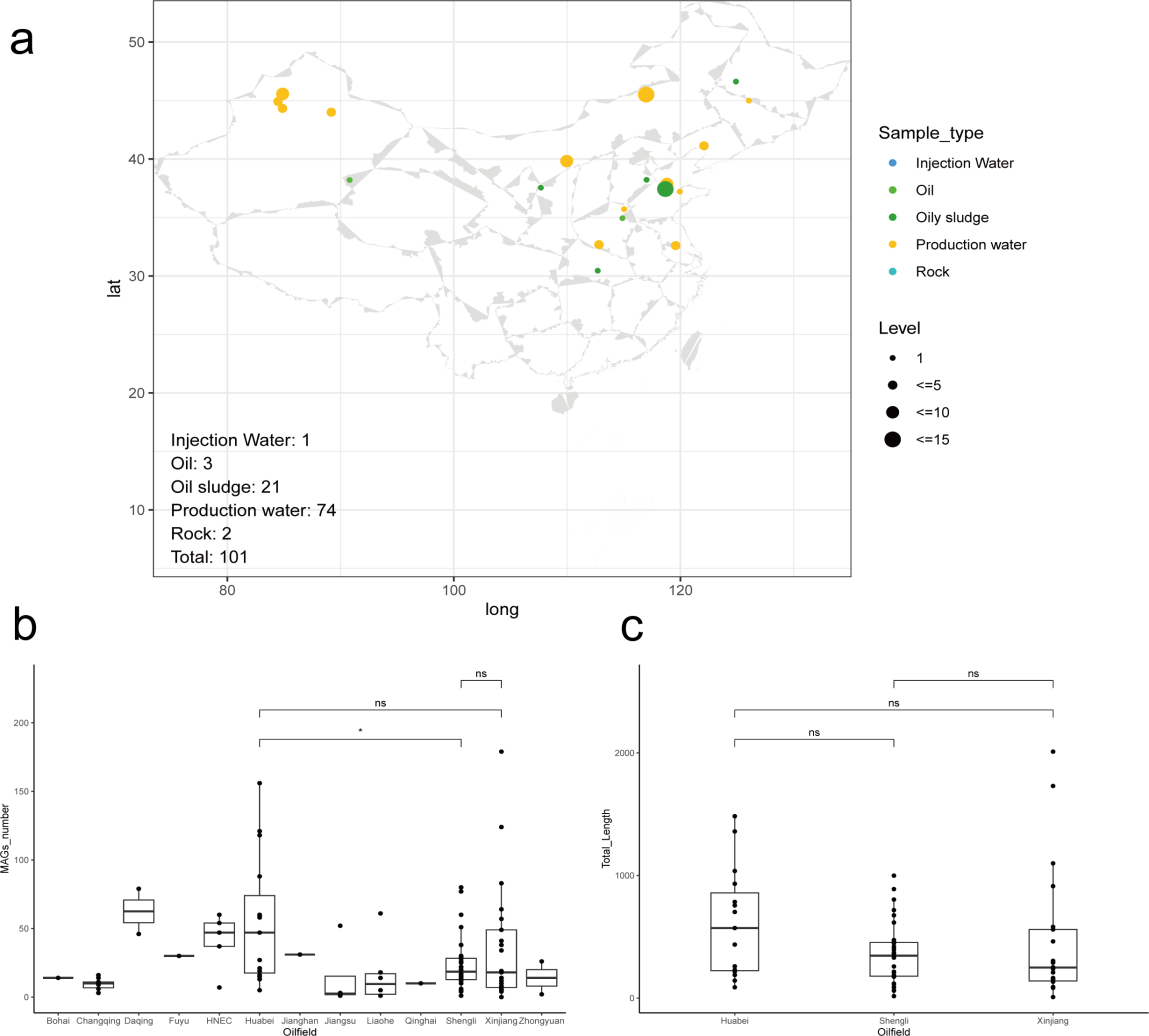
Information on 101 samples. (**a**) Sampling site distribution map. (**b**) Boxplot of MAG numbers obtained from different samples. *t*-test was used for significance test, “ns” means a *P*-value > 0.05, and “*” means a *P*-value ≤ 0.05 and *P*-value > 0.01. (**c**) Boxplot of assembly size for Huabei, Shengli, and Xinjiang samples. The statistical significance test method was the same as above.

In this study, we collected 101 publicly available samples from 13 oil fields across China (Fig. 1a; Tables S1 and S2). A total of 3,057 medium- and high-quality MAGs (completeness ≥50%; contamination ≤10%) were reconstructed. On average, 30 MAGs were obtained per sample (Fig. 1b). Across the 3,057 MAGs, a total of 8,514,112 coding proteins were functionally annotated, with an average of 2,785 coding genes per MAG. A production water sample from Xinjiang oilfields (Ref5_s24) yielded 179 MAGs, the highest number among all samples. Conversely, another production water sample from Xinjiang Oilfields (Ref4_s5) produced no MAGs, the lowest in the data sets. The assembly size of Ref4_s5 was 8.34 Mb, the smallest across all samples, explaining the absence of MAGs. While assembly sizes showed no significant differences among Huabei, Shengli, and Xinjiang oilfields, Huabei samples yielded notably more MAGs (Fig. 1b and c).

### Microbial distribution in China’s oilfields

**Fig 2:**
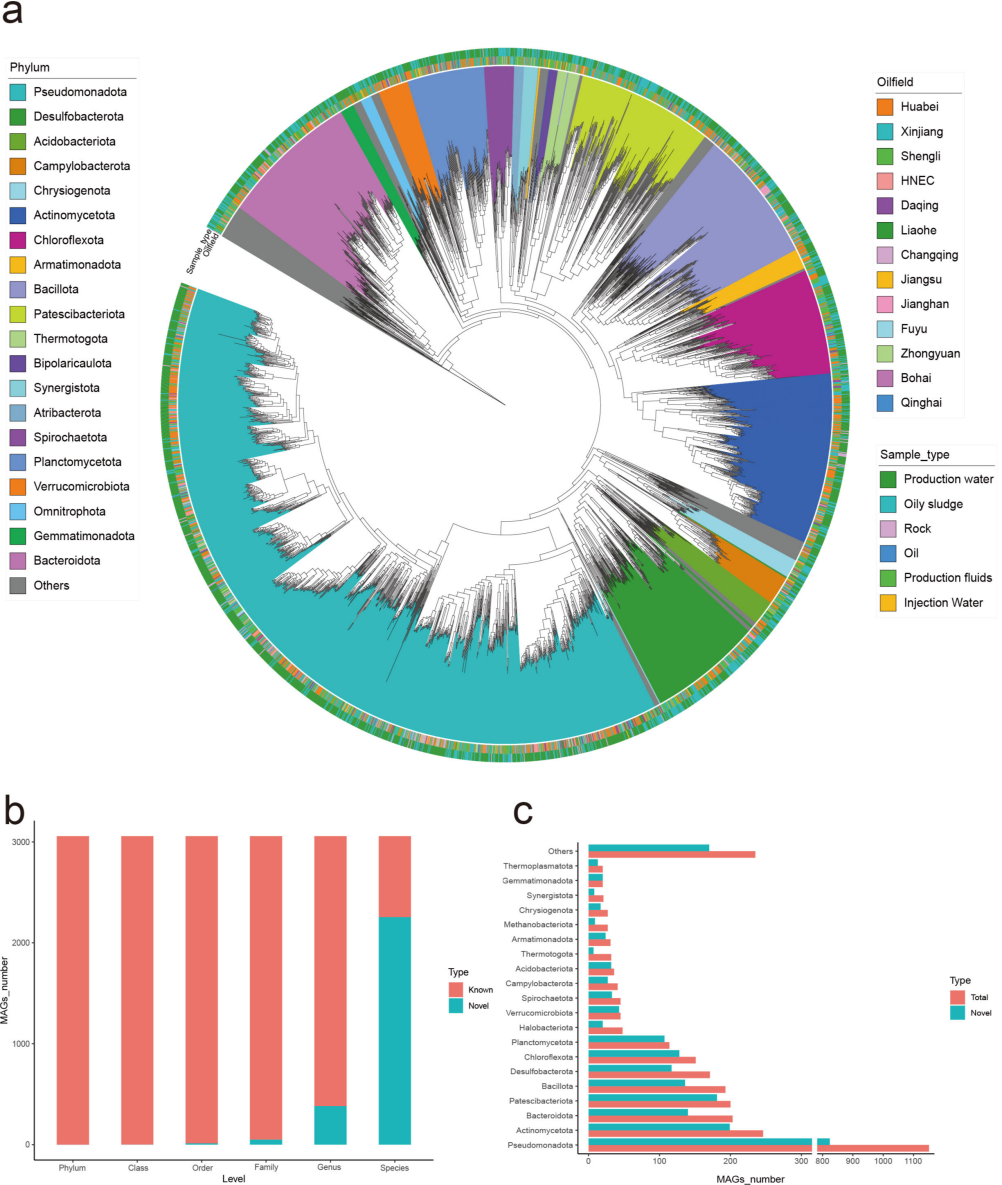
Taxonomic annotation information. (**a**) Phylogenetic tree of bacterial MAGs. Branch colors indicate different phyla; inner ring: sample source; outer ring: sample type. (**b**) Bar chart of novel MAG counts at different taxonomic levels. (**c**) Bar chart of novel MAG counts for top 20 genera.

The 3,057 MAGs were annotated against the GTDB v226 database, spanning 67 phyla and 1,319 genera (Fig. 2a; Fig. S1 and Table S3). Pseudomonadota, Actinomycetota, and Bacteroidota were the top three phyla by MAG count, while *Brevundimonas*, *Phenylobacterium,* and *Devosia* (within Pseudomonadota) ranked highest at the genus level. Among the 122 archaeal MAGs identified, the majority were associated with methane metabolism—primarily represented by the classes Methanobacteria, Methanosarcinia, and Methanomicrobia, with additional members belonging to the phylum Thermoplasmatota. Notably, 54 of these archaeal MAGs originated from the Shengli samples, consistent with previous reports indicating higher archaeal community diversity in Shengli samples (51). Notably, 2,255 MAGs (73.77%) lacked species-level annotations (Fig. 2b). Among the top 20 genera, >70% of MAGs were taxonomically uncharacterized at the species level. For genera *UBA9382*, *Ramlibacter*, *Hyphomicrobium*, and *Immundisolibacter*, more than 90% of MAGs represented novel species (Fig. 2c), highlighting vast unexplored microbial diversity in oil environments and the breakthrough potential of this data set in revealing “microbial dark matter.”

**Fig 3:**
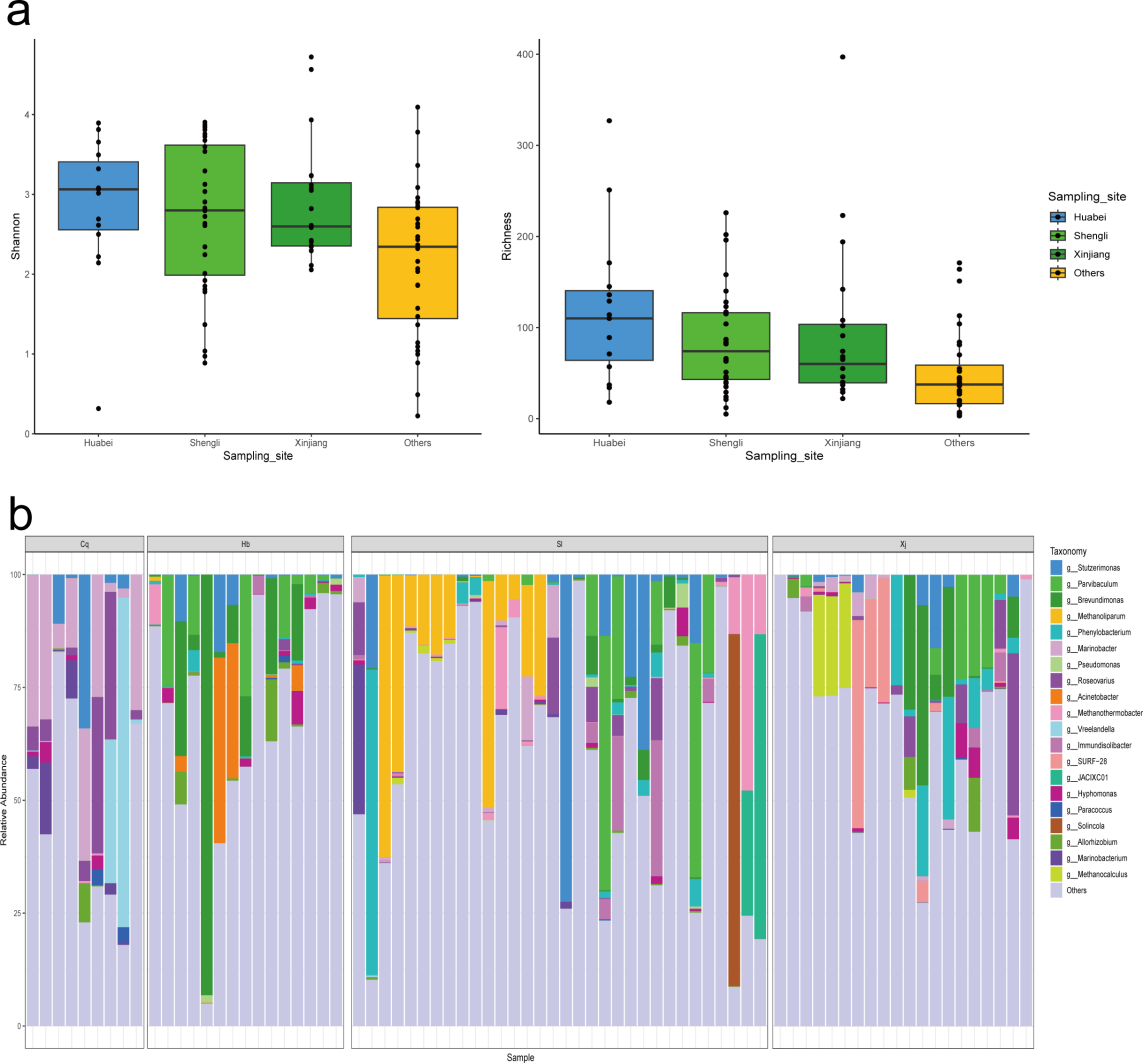
Species abundance information. (**a**) Alpha diversity results (left: Shannon index boxplot; right: richness boxplot). (**b**) Bar chart of species composition for four oilfield samples (Cq: Changqing, Hb: Huabei, Sl: Shengli, and Xj: Xinjiang).

To further investigate the microbial distribution across oilfields, we calculated the relative abundance and alpha diversity (see Methods). Shannon index and richness values indicated higher species diversity in Huabei, Shengli, and Xinjiang samples compared to others, with Huabei exhibiting the most complex and stable community structure (Fig. 3a). Additionally, some microbes showed oilfield-specific distributions (Fig. 3b; Fig. S2 and Table S4). For example, *Methanoliparum* and *Methanothermobacter* were predominantly abundant in Shengli’s oily sludge samples. The other methanogen, *Methanocalculus,* was detected only in Xinjiang samples. *Marinobacter* was ubiquitous in Changqing oilfields but rare elsewhere. *Brevundimonas* and *Parvibaculum* were widespread across Huabei, Shengli, and Xinjiang samples.

### Planctomycetia: a lineage with large genomes

**Fig 4:**
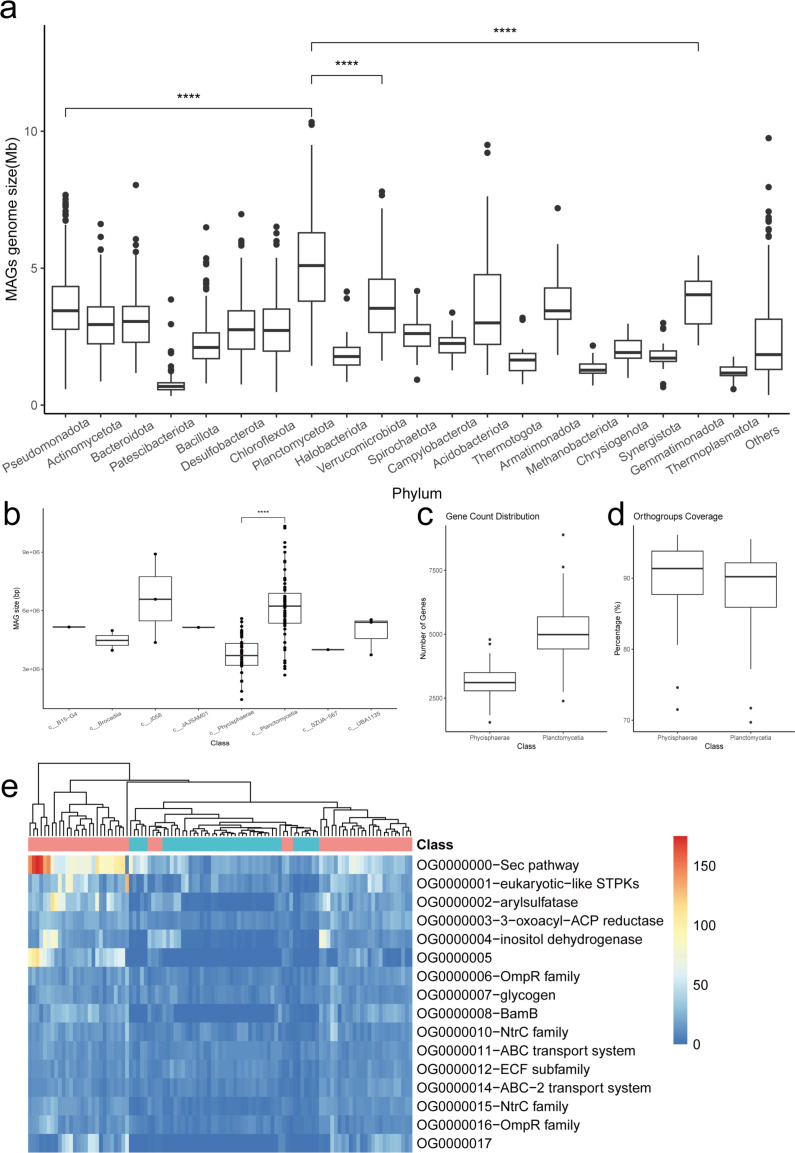
Planctomycetia-class MAG information. (**a**) Boxplot of genome size for MAGs of top 20 phyla. *t*-test was used for significance test, and “****” means a *P* value ≤ 0.0001. (**b**) Boxplot of genome size for Planctomycetota phylum MAGs. The statistical significance test method was the same as above. (**c**) Boxplot of gene counts for Planctomycetia and Phycisphaerae classes. (**d**) Boxplot of gene coverage for Planctomycetia and Phycisphaerae classes. (**e**) Heatmap of gene counts in Large_orthogroups. Red represents MAGs of the class Planctomycetia, while blue represents MAGs of the class Phycisphaerae.

Among the 3,057 MAGs, genome sizes ranged from 3.33 Mb to 10.33 Mb. Eight MAGs exceeded genome sizes of 8 Mb, all belonging to the class Planctomycetia (phylum Planctomycetota). Comparative analysis of the top 20 phyla confirmed that Planctomycetota MAGs were significantly larger (Fig. 4a). Further comparison within Planctomycetota revealed that Planctomycetia (59 MAGs) and Phycisphaerae (44 MAGs) were the dominant classes, with Planctomycetia genomes being significantly larger than Phycisphaerae (Fig. 4b).

To assess whether MAG quality influenced genome size, we analyzed N50, QS (QS = completeness – 5 × contamination), and size relationships (Fig. S3 and S4). Planctomycetia and Phycisphaerae exhibited similar distributions of N50 and QS, indicating comparable MAG quality. At equivalent N50 or QS values, Planctomycetia genomes remained larger, confirming that quality did not drive size differences.

We analyzed gene counts in 103 MAGs (59 Planctomycetia + 44 Phycisphaerae) and clustered genes into OGs using OrthoFinder v2.5.5. Planctomycetia MAGs harbored significantly more genes than Phycisphaerae (Fig. 4c), explaining their larger genomes. The 103 MAGs contained 436,764 genes, of which 388,838 (89%) clustered into 21,330 OGs. Over 70% of genes per MAG were assignable to OGs (Fig. 4d; Table S5).

OGs were filtered and categorized as follows: **a. Planctomycetia_Phycisphaerae_common**, 248 OGs present in >90% of MAGs from both classes; **b. Planctomycetia_Phycisphaerae_specific**: 467 OGs present in >90% of MAGs from one class but not the other; **c. Large orthogroups**: 16 OGs with >10 gene copies per MAG on average (Table S6). Heatmaps of these OGs revealed that Planctomycetia MAGs consistently contained higher gene copy numbers in specific OGs than Phycisphaerae, but not vice versa (Fig. 4e). Furthermore, genes within these OGs predominantly function in energy and nutrient metabolism, as well as signal transduction. Examples include ATP hydrolysis (ABC transport system), glycogen metabolism, fatty acid synthesis (3-oxoacyl-ACP reductase), carbon metabolism (inositol dehydrogenase), the general secretion pathway (Sec pathway), and two-component systems (OmpR family and NtrC family). Consequently, compared to Phycisphaerae, species within the Planctomycetia class exhibit higher metabolic activity and require a greater number of functional genes. Additionally, we found that Planctomycetia possesses a higher abundance of eukaryotic-like serine/threonine protein kinases (STPKs), and the genes encoding these kinases were likely acquired via horizontal gene transfer from eukaryotes. In summary, we propose that the combined effects of functional gene redundancy and horizontal gene transfer contribute to the observed increase in genome size within the Planctomycetia class.

### Alkane metabolism gene mining

Through HMMER alignment and redundancy reduction, we identified 38 candidate AcrA genes, alongside 910 AssA, 478 BcrB, and 395 BcrC genes. Subsequent phylogenetic screening yielded 68 high-confidence alkane metabolism-related genes (3 AcrA, 17 AssA, 21 BcrB, and 27 BcrC; Table S7).

**Fig 5:**
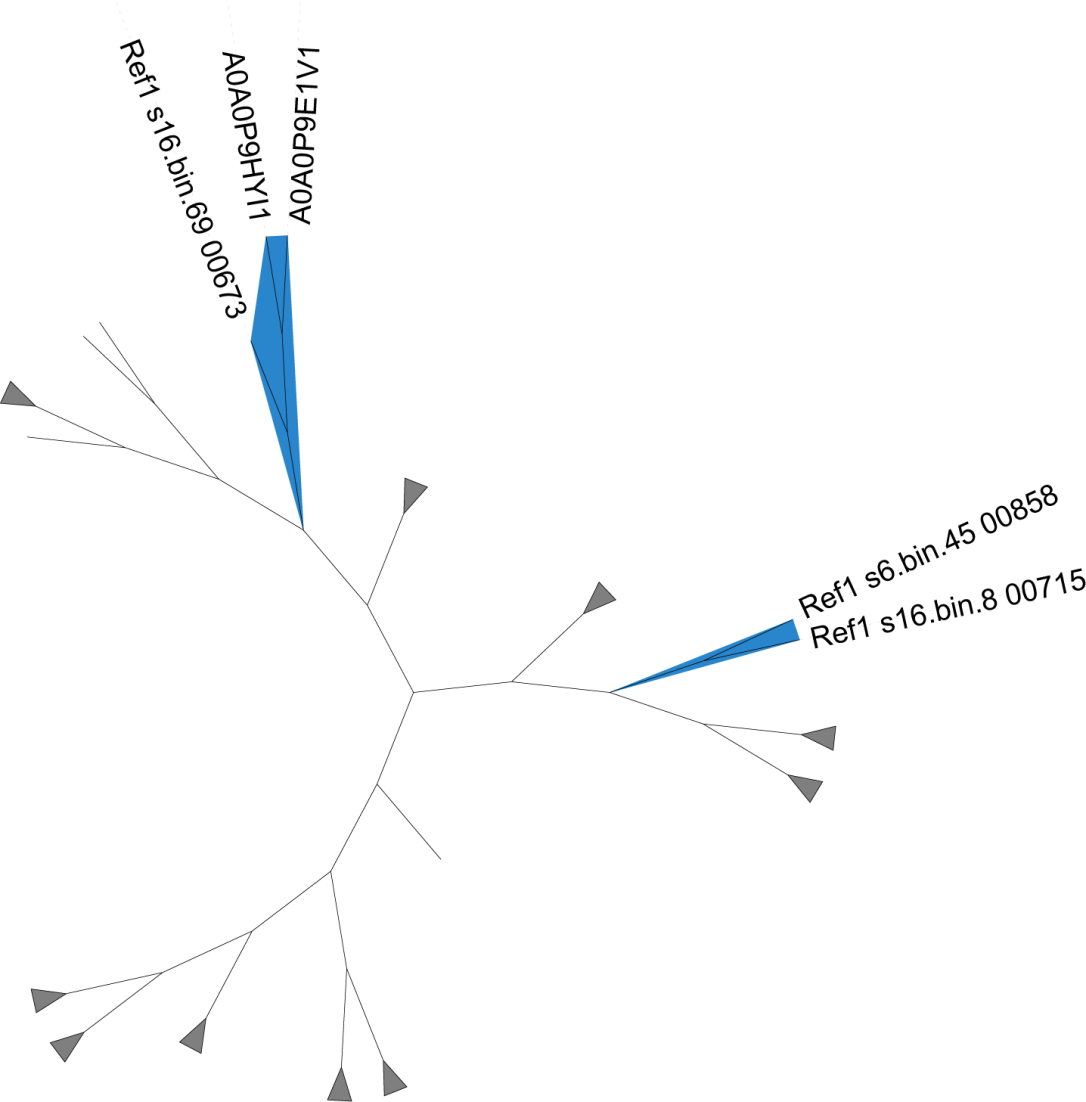
Phylogenetic tree of AcrA candidate genes and reference genes. Branches with the blue background indicate screened AcrA genes.

Candidate AcrA genes primarily originated from the classes Halobacteriota, Methanobacteriota, and Thermoplasmatota. Notably, among four Methanoliparum MAGs in our data sets, three contained candidate AcrA genes (Ref1_s6.bin.45_00858, Ref1_s16.bin.8_00715, and Ref1_s16.bin.69_00673). A phylogenetic tree was constructed using reference genes from the KEGG database (KO: K00399) and the 38 candidate AcrA genes (Fig. 5). Within this tree, Ref1_s6.bin.45_00858 and Ref1_s16.bin.8_00715 formed a distinct cluster. Ref1_s16.bin.69_00673 clustered with reference sequences A0A0P9HYI1 and A0A0P9E1V1, both derived from the phylum Bathyarchaeota (classified as class Bathyarchaeia in GTDB v226). Bathyarchaeota members have been implicated in methane metabolism or interactions with methanogens (52, 53). Within our data set, an MAG assigned to the class Bathyarchaeia (Ref5_s8.bin.147) was identified; however, no candidate AcrA genes were detected in this genome. The remaining candidate AcrA genes did not cluster with Methanoliparum-derived sequences, suggesting they represent conventional McrA homologs.

Seventeen candidate AssA genes clustered with reference sequences (Fig. S5), 65% of which belonged to the phylum Desulfobacterota, consistent with prior reports (12). Similarly, 21 BcrB and 27 BcrC genes clustered with their respective reference sequences (Fig. S6 and S7). These sequences predominantly originated from the phyla Pseudomonadota and Planctomycetota, aligning with reference sequences from AnnoTree (47).

### Secondary metabolite biosynthetic gene clusters and novel antimicrobial peptide discovery

**Fig 6:**
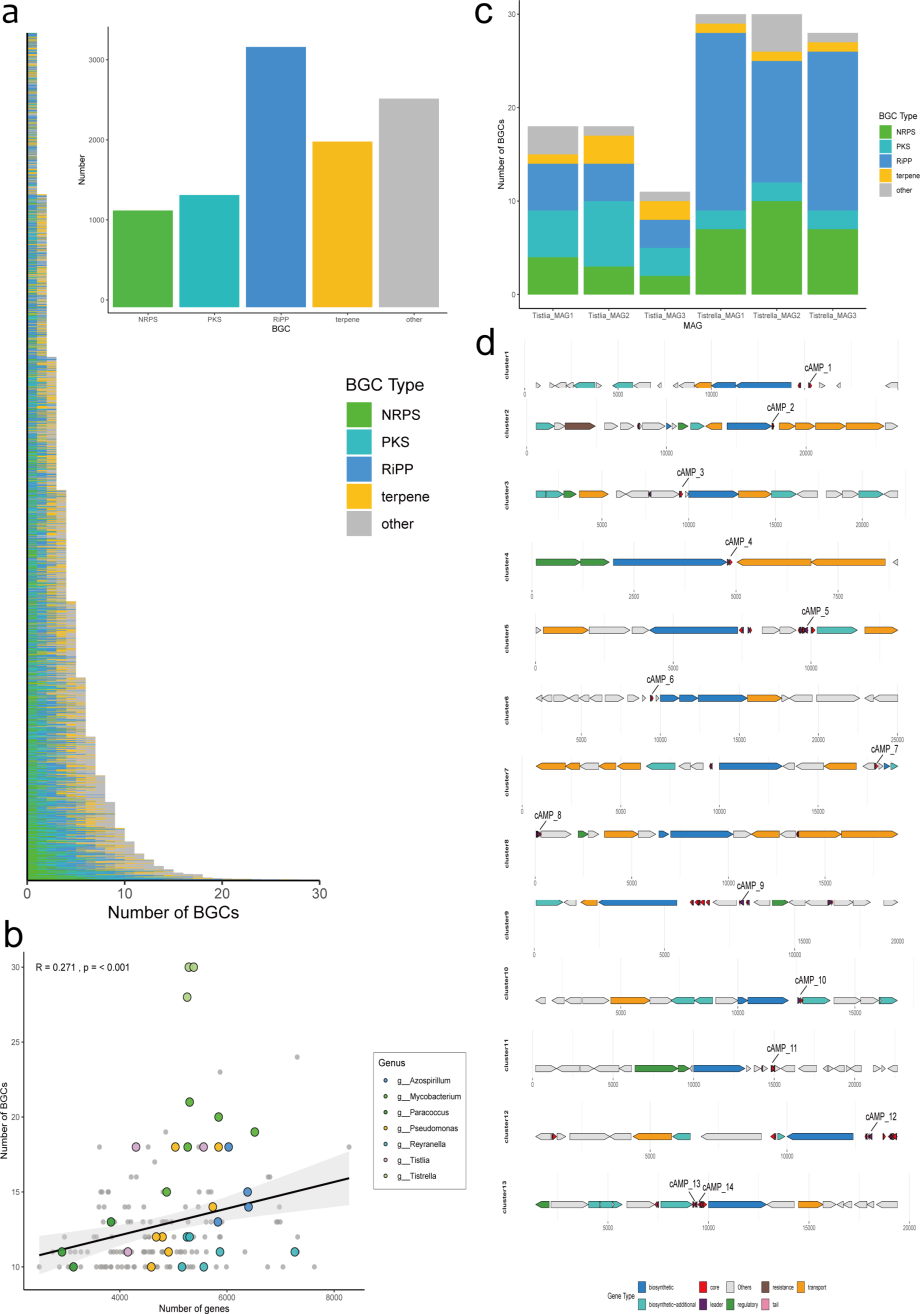
BGC and cAMP information. (**a**) Bar chart of BGC type counts per MAG (y-axis: MAGs; top-right bar: total BGC counts per type). (**b**) Scatter plot of gene counts vs BGC counts for MAGs with abundant BGCs (colors indicate genera). (**c**) Bar chart of BGC counts for *Tistrella* and *Tistlia*. (**d**) Gene distribution map of 13 BGCs containing cAMPs.

BGCs within MAGs were identified using antiSMASH. A total of 10,168 BGCs were detected across 2,580 MAGs (Table S8). Ribosomally synthesized and post-translationally modified peptides (RiPPs) constituted the most abundant class (3,251), followed by terpenes (2,069), polyketide synthases (PKS; 1,401), and NRPSs (1,208) (Fig. 6a). Analysis of BGCs per MAG revealed that 94% of MAGs contained fewer than 10 BGCs. Two MAGs harbored 30 BGCs each, predominantly RiPPs (Fig. 6a). For MAGs with ≥10 BGCs, no significant correlation was observed between BGC count and total gene count (R = 0.271). Interestingly, certain genera consistently exhibited high BGC numbers. For example, all three MAGs assigned to the genus *Tistrella* (phylum Pseudomonadota, class Alphaproteobacteria) contained >25 BGCs. Similarly, all three MAGs of the related genus *Tistlia* contained >10 BGCs (Fig. 6b). Within *Tistrella* MAGs, RiPPs dominated the BGC profile, whereas *Tistlia* MAGs primarily contained RiPPs and PKSs (Fig. 6c).

Based on BGC analysis, we identified 14 cAMPs from 13 lanthipeptide BGCs (Table S9). These peptides ranged from 10 to 59 amino acids in length and predominantly derived from the classes Alphaproteobacteria (6 cAMPs) and Gammaproteobacteria (3 cAMPs) within the phylum Pseudomonadota. None of these 14 cAMPs are cataloged in the Antimicrobial Peptide Database 6 (APD6) (54), representing novel cAMPs identified for the first time in petroleum microbiomes. The cAMPs were consistently located adjacent to biosynthetic genes within their BGCs (Fig. 6d).

## DISCUSSION

Substantial variation in sequencing depth was observed across samples due to diverse data sources. Sequencing depth, assembly size, and MAG yield demonstrated positive correlations, with a particularly significant association between assembly size and MAG count (Fig. S8). Thus, for petroleum microbiome metagenomic studies, maximizing the sequencing depth is recommended where feasible to ensure robust downstream analyses.

From 15 Huabei samples, we recovered 823 MAGs, compared to 758 MAGs from 32 Shengli samples. Although total assembly sizes showed no significant difference between fields (Fig. 1c), three Huabei samples yielded assemblies >1,000 Mb, each producing >100 MAGs per sample—a pattern absent in Shengli samples. This accounts for the higher MAG recovery from Huabei oilfields. Taxonomic analysis revealed 596 genera in Huabei oilfields versus 533 in Shengli oilfields, with only 106 genera overlapping (Fig. S9), indicating substantial biogeographic divergence. While previously noted, the drivers of this disparity warrant further investigation integrating geochemical parameters.

The phylum Gemmatimonadota was exclusively detected in production water and oily sludge (Fig. 2a), while previous studies reported its presence in soil, freshwater, wastewater, marine, and sediment environments (55–59). Gemmatimonadota was phylogenetically classified into five classes, with the third class, Longimicrobia (corresponding to Longimicrobiales in GTDB v226), primarily found in oil and gas hydrates (60, 61), consistent with our results, suggesting Longimicrobiales may be an oil-specific microbe.

Our data set contained four *Methanoliparum* MAGs, three originating from Shengli oilfields—consistent with *Methanoliparum’s* initial isolation from this field (17). Shengli oilfields also harbored higher archaeal abundance, suggesting enhanced biodegradation potential. 16S rRNA studies indicate *Methanoliparum’s* environmental plasticity, having been detected in diverse settings including a biodegraded Nigerian reservoir (62) and a sulfur-rich Canadian offshore reservoir (63), highlighting its bioremediation utility for petroleum-contaminated sites.

AcrA, a key alkane-degrading gene in *Methanoliparum* (64), prompted identification of two candidate AcrA homologs from Bathyarchaeota in Swiss-Prot. Bathyarchaeota thrive globally in anaerobic niches—hot springs (65), sediments (66, 67), acid-sulfate springs (68), animal guts (69), and bioreactors (70)—playing crucial roles in carbon cycling (17). While Evans (71) reported Mcr (indicating methanogenesis) in two Bathyarchaeota MAGs (BA1 and BA2), recent work found no Mcr in strains ILS200 and ILS300 (72), aligning with the results of our Bathyarchaeia MAG (Ref5_s8.bin.147).

To resolve this discrepancy, we annotated all five MAGs using GTDB v226. BA1 and BA2 clustered within Bathyarchaeaceae, whereas ILS200 belonged to Bathycorpusculaceae, and ILS300 clustered with Ref5_s8.bin.147 to families WUQV01 and TCS64 (Table S10). Phylogenetic analysis revealed closer evolutionary proximity among ILS200, ILS300, and Ref5_s8.bin.147 (Fig. S10), suggesting methane metabolism may be restricted to specific lineages like Bathyarchaeaceae.

Three *Tistrella* MAGs in our data set contained the highest number of BGCs. *Tistrella* produces didemnin—an NRPS with anticancer and antiviral properties (73–75). Initially isolated from marine environments (76), didemnin-producing *Tistrella* has since been found in wastewater (77), heavy metal-contaminated soil (78), and the Red Sea (79). Our *Tistrella* MAGs from Xinjiang, Changqing, and Daqing samples represent its first recovery from oil reservoirs. These MAGs harbored 7–10 NRPS-type BGCs, ranking among the highest NRPS-rich genera observed, indicating potential for synthesizing bioactive peptides.

Three cAMP prediction models, previously validated in human gut and marine microbiomes (21, 22), identified only 14 cAMPs in our data set. This limited yield likely reflects our data set size. We anticipate increased cAMP discovery with expanded sampling. All cAMPs in our data set were located near biosynthetic genes (Fig. 6d). These biosynthetic genes are responsible for post-translational modifications of precursor peptides, enabling functional diversification for antimicrobial, anticancer, and other bioactivities (80).

This study analyzed 101 metagenomes from 13 Chinese oilfields, generating 3,057 medium- and high-quality MAGs. Notably, 73.77% represent novel species. We observed gene family expansion in Planctomycetia, correlating with increased genome size. Additionally, 68 anaerobic hydrocarbon biodegradation genes were identified. This work reports the first reservoir-derived *Tistrella* MAGs (producers of anticancer/antiviral compounds) and 14 novel cAMPs. Petroleum reservoirs constitute a rich microbial treasure trove. Enhanced research promises advances not only in oil recovery but also in biosynthesis and biomedical applications.

## Data Availability

All raw data were obtained from public databases, and detailed information is provided in Table S1. The 3,057 MAGs have been uploaded to Figshare and are available for viewing and downloading via https://doi.org/10.6084/m9.figshare.31337758.

## SUPPLEMENTAL MATERIAL

Supplemental figures aem.00392-26-s0001.docx Fig. S1 to S10.

Supplemental tables aem.00392-26-s0002.xlsx Tables S1 to S11.

ASM does not own the copyrights to Supplemental Material that may be linked to, or accessed through, an article. The authors have granted ASM a non-exclusive, world-wide license to publish the Supplemental Material files. Please contact the corresponding author directly for reuse.
