# Supplementary material for: Metagenomic mining reveals extensive novelty, enhanced biodegradation potential, and untapped biosynthetic capacity in Chinese oilfield microbiomes
Source: Appl Environ Microbiol. 2026 Apr 1;92(4):e00392-26. doi: 10.1128/aem.00392-26 (PMC13101482; doi:10.1128/aem.00392-26)
Supplement: Supplemental figures — Fig. S1 to S10. [file aem.00392-26-s0001.docx]

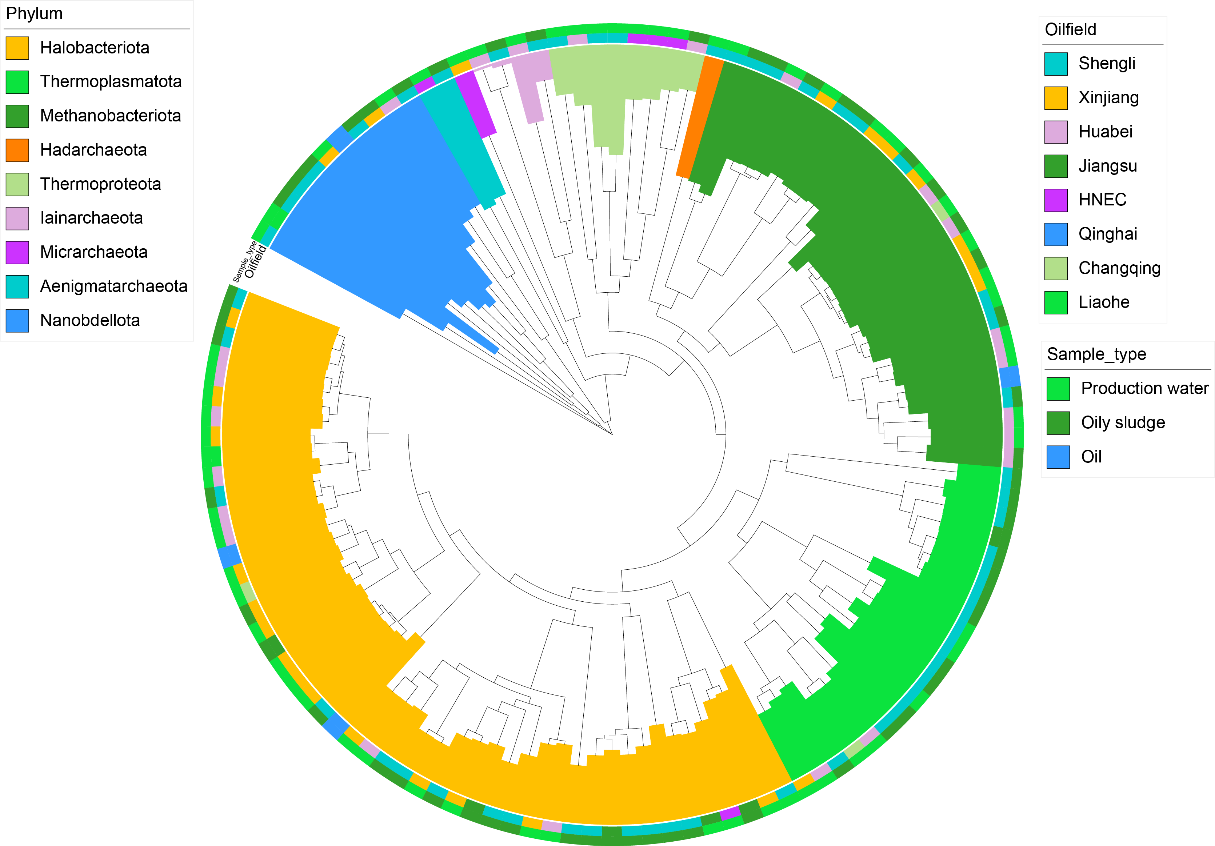


**Supplementary Fig. 1** Phylogenetic tree of archaeal MAGs. Branch colors indicate different phyla; inner ring: sample source; outer ring: sample type.


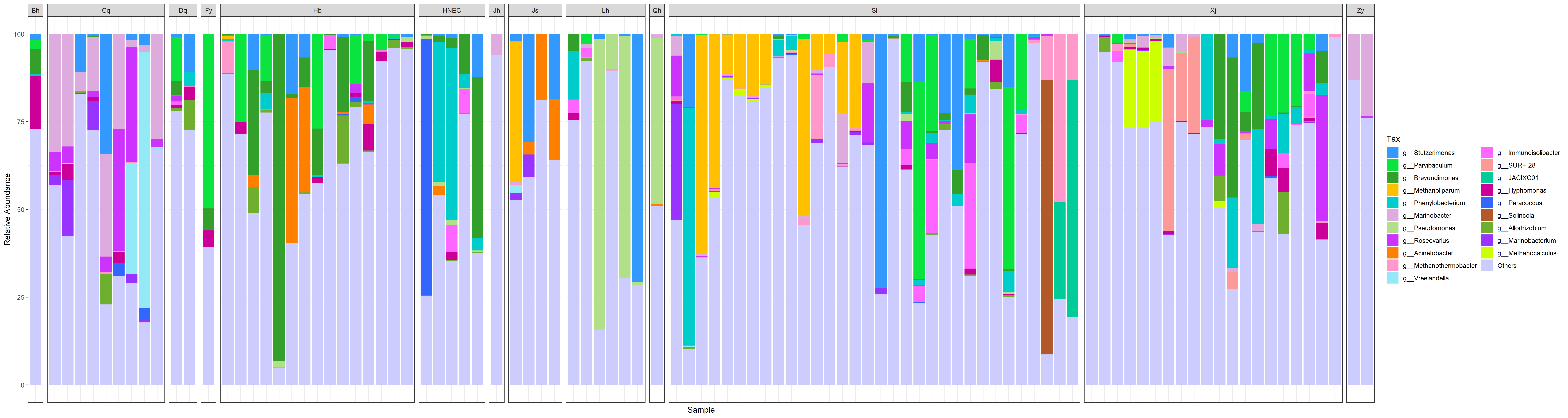


**Supplementary Fig. 2** Bar chart of species composition for 101 samples (Bh: Bohai, Cq: Changqing, Dq: Daqing, Fy: Fuyu, Hb: Huabei, Jh: Jianghan, Js: Jiangsu, Lh: Liaohe, Qh: Qinghai, Sl: Shengli, Xj: Xinjiang, Zy: Zhongyuan).


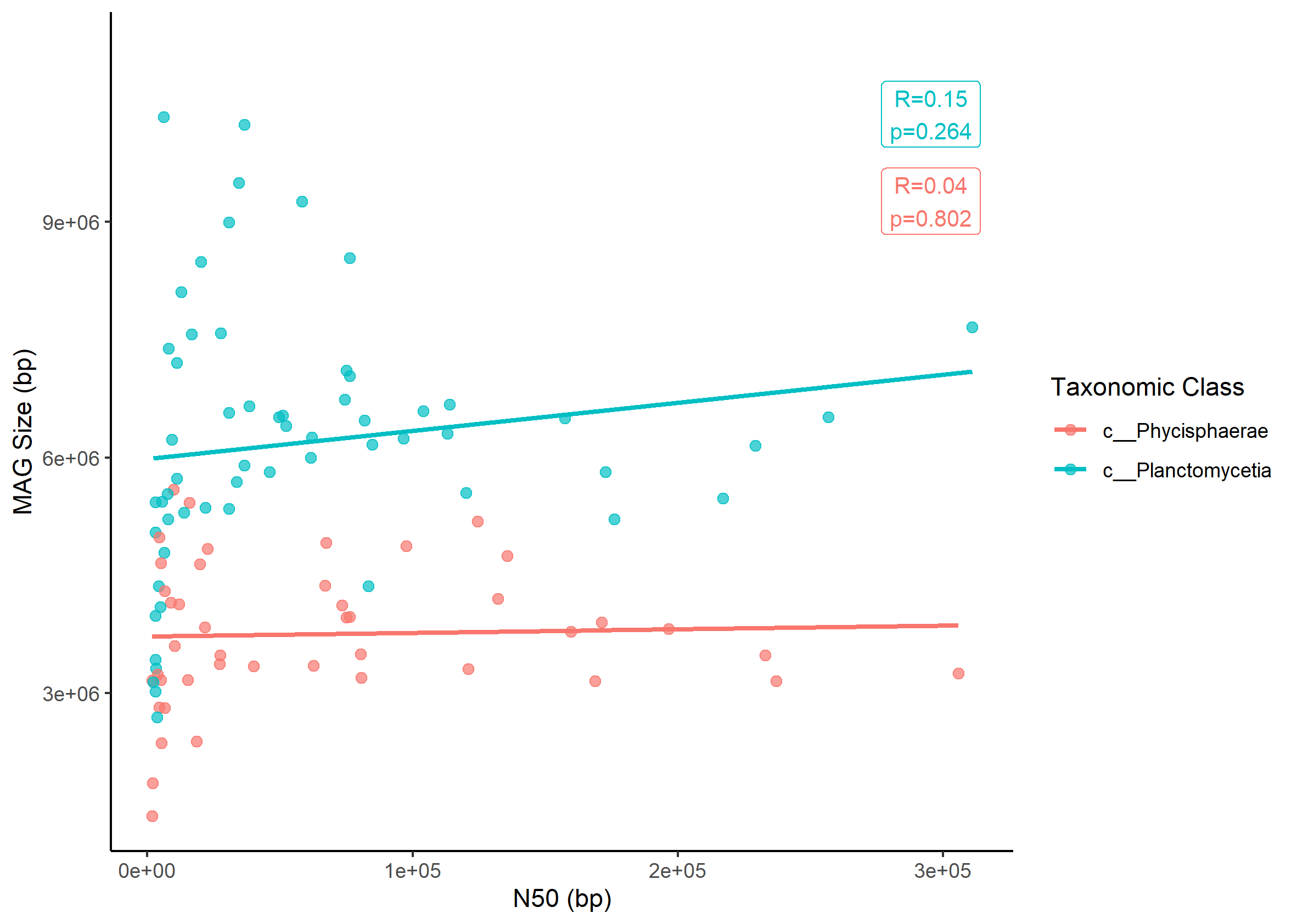


**Supplementary Fig. 3** Scatter plot of N50 vs. MAG size for Planctomycetia and Phycisphaerae classes.


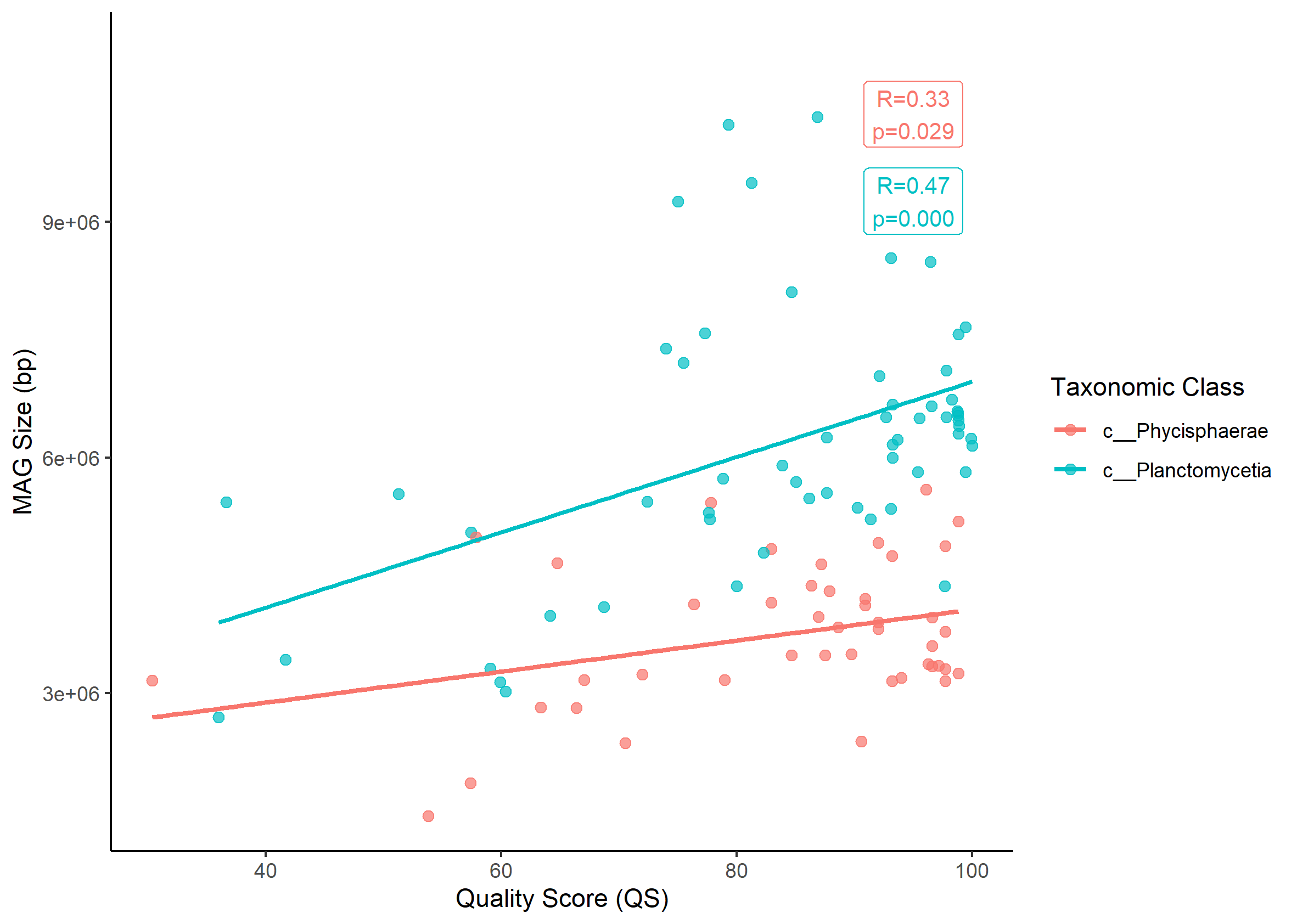


**Supplementary Fig. 4** Scatter plot of quality score vs. MAG size for Planctomycetia and Phycisphaerae classes.


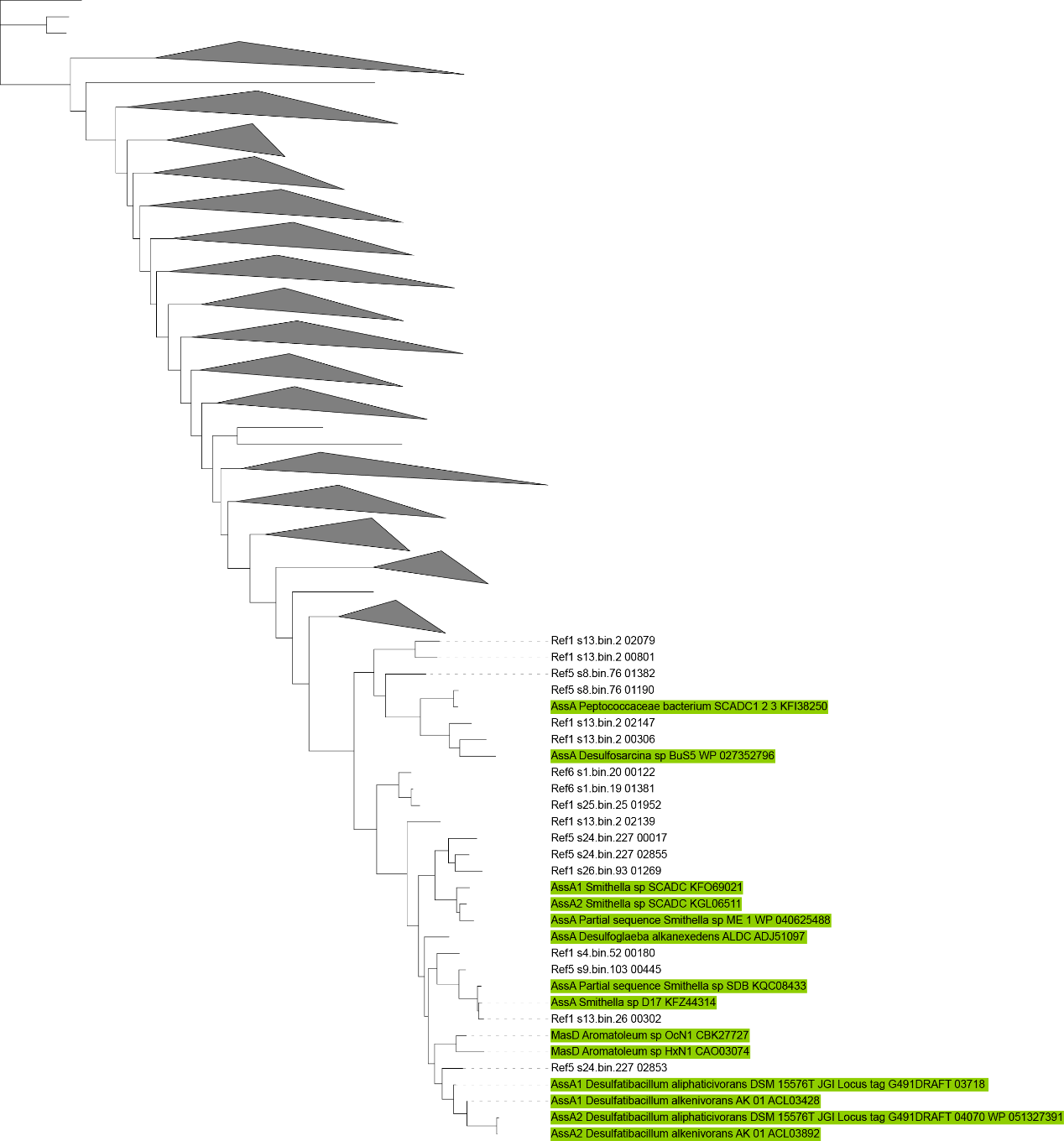


**Supplementary Fig. 5** Phylogenetic tree of AssA candidate genes and reference genes. Genes with green background are reference genes.


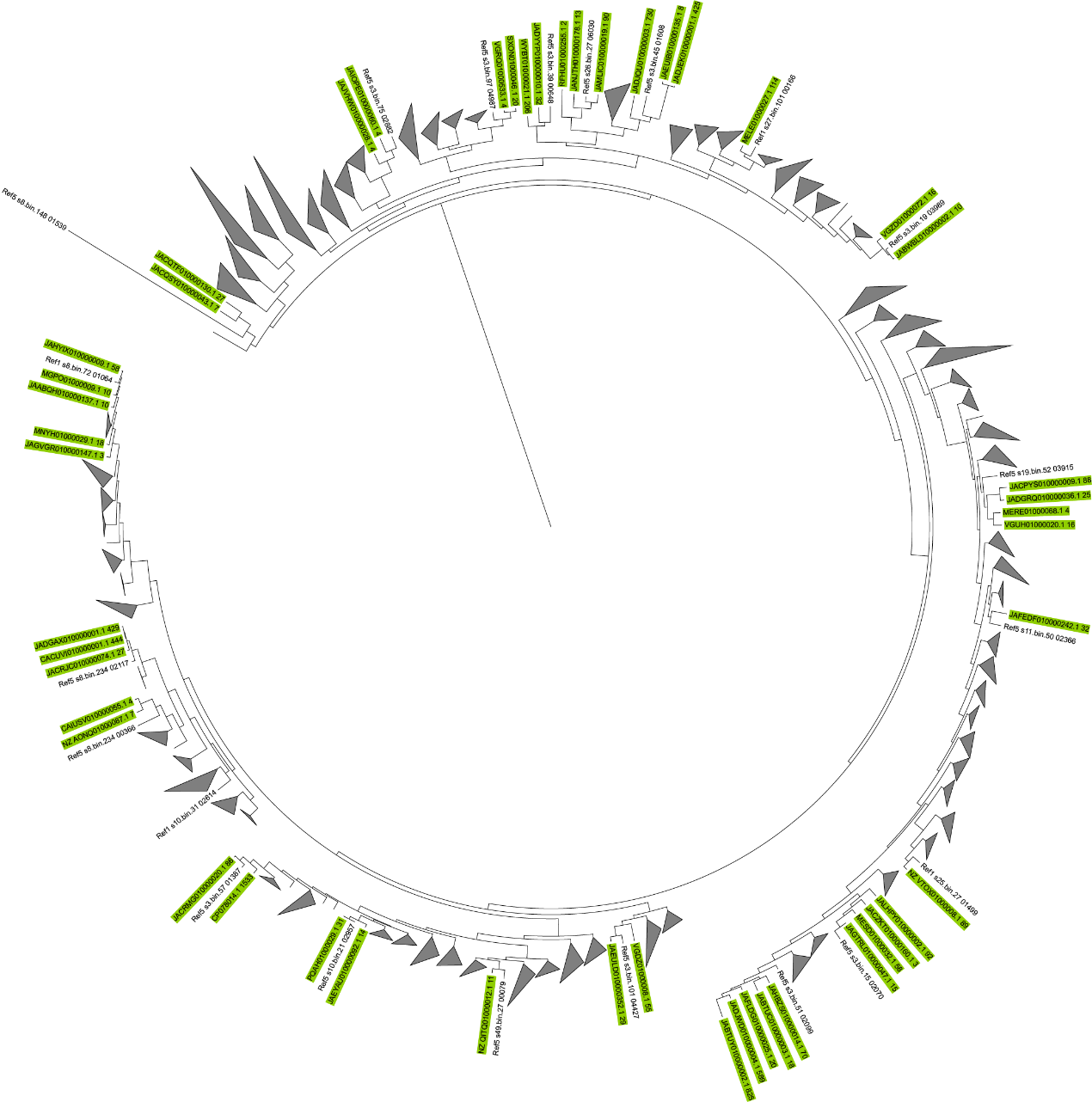


**Supplementary Fig. 6** Phylogenetic tree of BcrB candidate genes and reference genes. Genes with green background are reference genes.


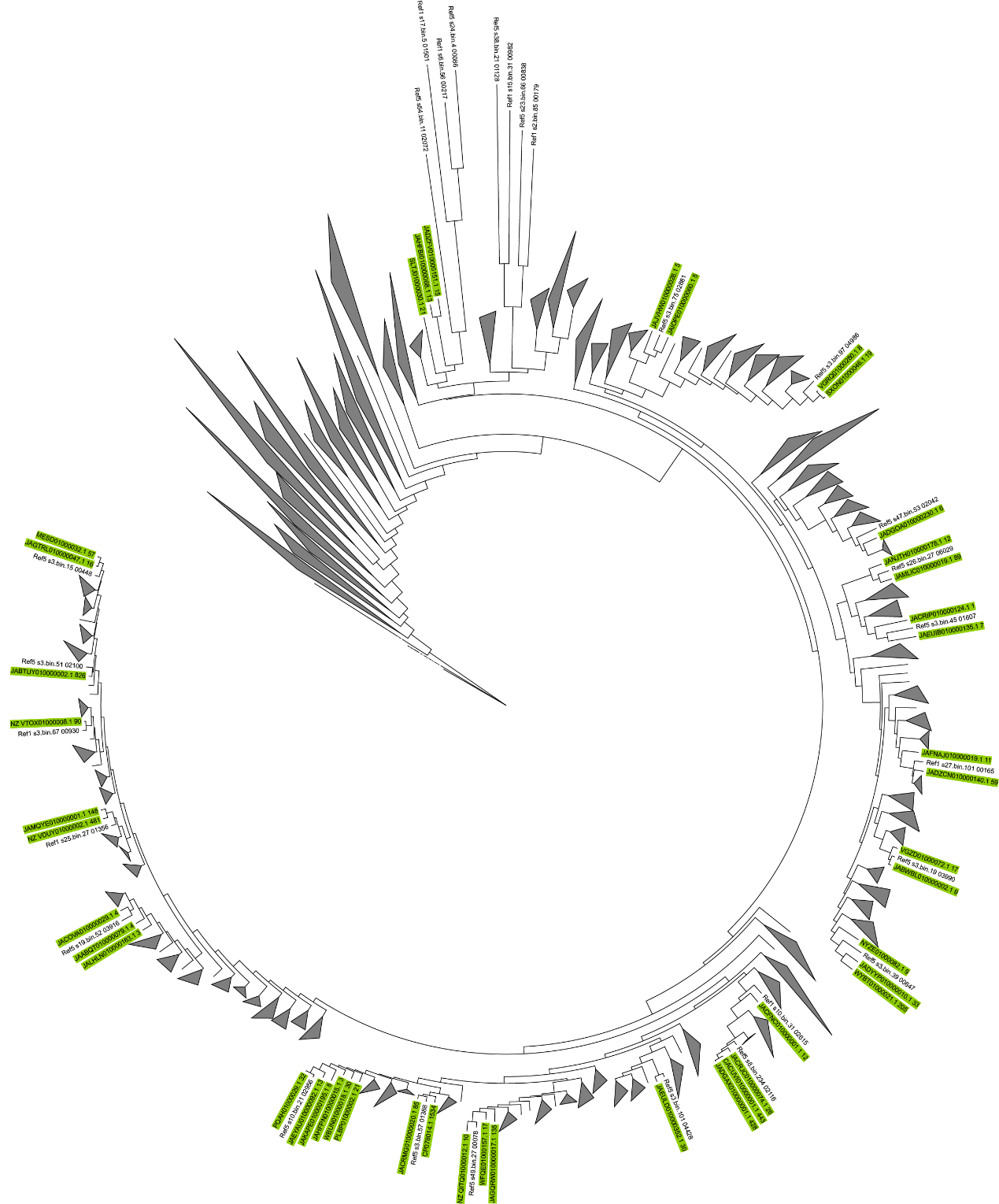


**Supplementary Fig. 7** Phylogenetic tree of BcrC candidate genes and reference genes. Genes with green background are reference genes.


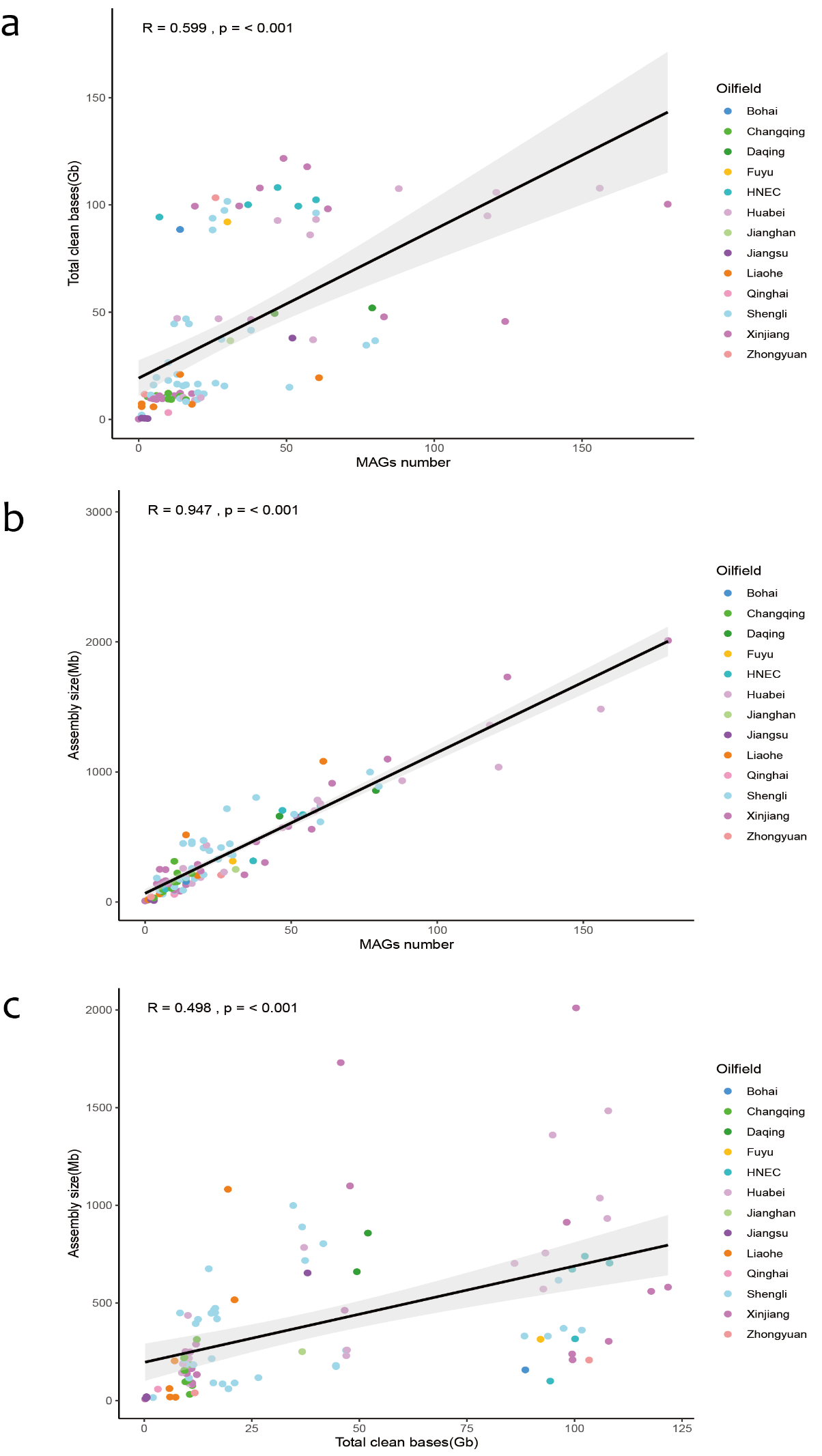


**Supplementary Fig. 8** Relationships among clean data, assembly size, and MAG counts. a. Scatter plot of clean bases vs. MAG counts. b. Scatter plot of assembly size vs. MAG counts. c. Scatter plot of clean bases vs. assembly size.


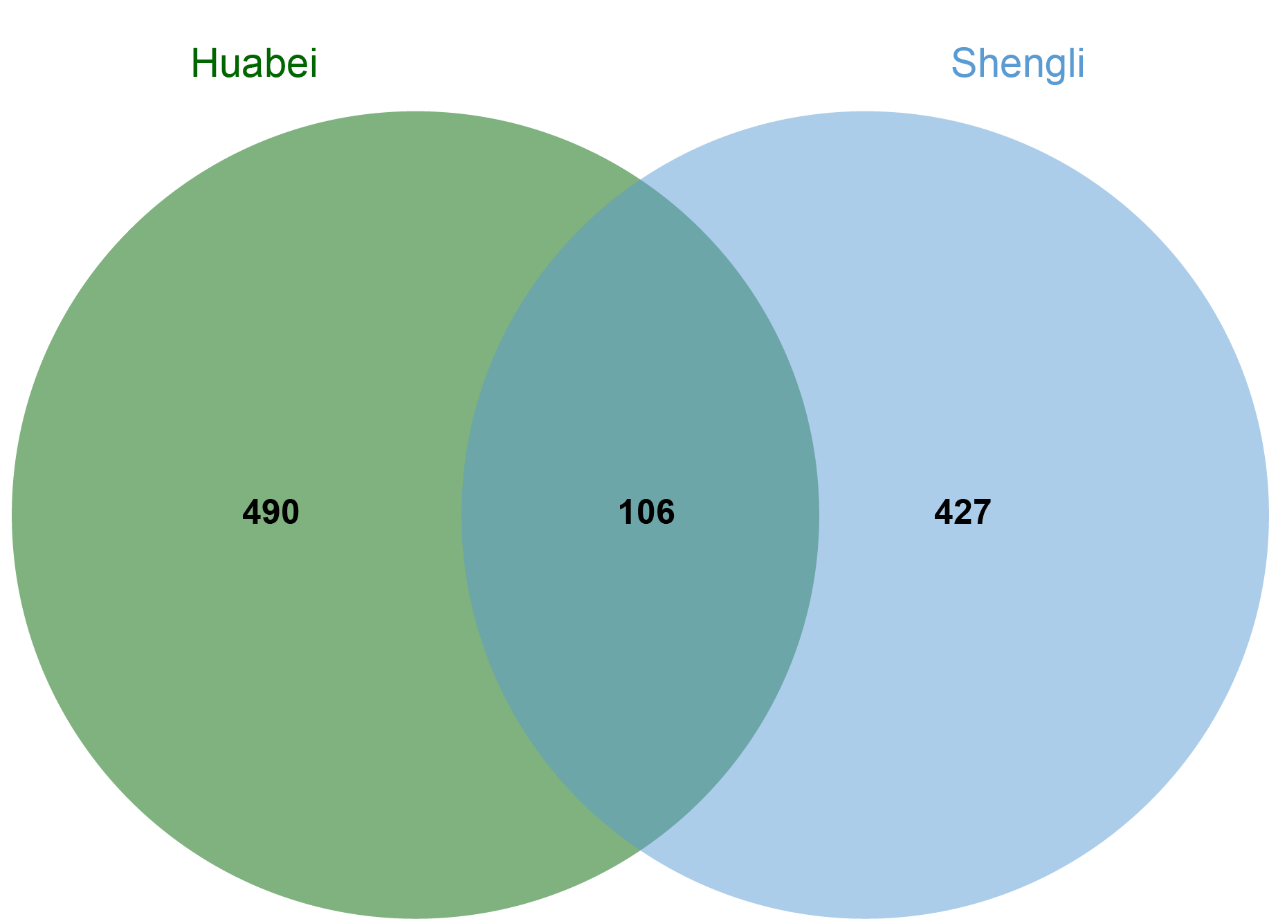


**Supplementary Fig. 9** Venn diagram of genus-level species counts in Huabei and Shengli oilfields.


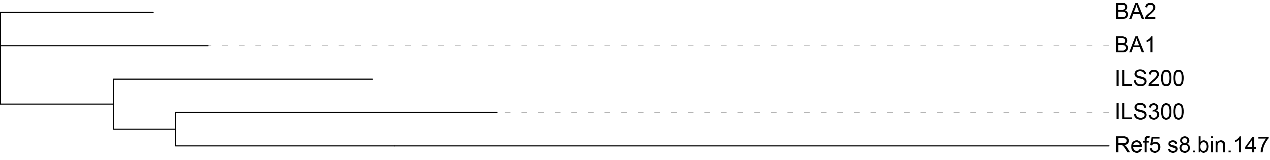


**Supplementary Fig. 10** Phylogenetic tree of 5 Bathyarchaeota MAGs.
